# Alcohol-Responsive Hyperkinetic Movement Disorders—a Mechanistic Hypothesis

**DOI:** 10.5334/tohm.560

**Published:** 2020-10-21

**Authors:** Steven J. Frucht, Giulietta M. Riboldi

**Affiliations:** 1 NYU Grossman School of Medicine, Division of Movement Disorders, New York, NY, US

**Keywords:** alcohol, GHB, sodium oxybate, tremor, myoclonus, dystonia

## Abstract

Patients with essential tremor, vocal tremor, torticollis, myoclonus-dystonia and posthypoxic myoclonus often benefit in a surprisingly rapid and robust manner from ingestion of a modest amount of alcohol (ethanol). Despite considerable investigation, the mechanism of ethanol’s ability to produce this effect remains a mystery. In this paper, we review the pharmacology of ethanol and its analogue GHB (or sodium oxybate), summarize the published literature of alcohol-responsive hyperkinetic movement disorders, and demonstrate videos of patients we have treated over the last fifteen years with either an ethanol challenge or with chronic sodium oxybate therapy. We then propose a novel explanation for this phenomenon—namely, that ingestion of ***modest*** doses of ethanol (or sodium oxybate) normalizes the aberrant motor networks underling these disorders. We propose that alcohol and its analogues improve clinical symptoms and their physiologic correlate by restoring the normal firing pattern of the major outflow pathways of the cerebellum (the Purkinje cells and deep cerebellar nuclei), We present evidence to support this hypothesis in animal models and in affected patients, and suggest future investigations to test this model.

## Background

Ethanol (EtOH) has long been known to exert a deleterious effect on the brain. The acute effects of EtOH ingestion include mild dizziness, decreased reaction time, dulled perception, tremor, myoclonus and ataxia. Chronic alcohol intoxication can also result in development of tolerance, dependence and psychiatric symptoms. Nevertheless, EtOH possesses a remarkable ability to improve the severity of specific hyperkinetic movement disorders. This effect may be obvious and profound, and presents a challenge to the treating physician who must balance the potential benefit from EtOH with the serious concerns of chronic EtOH administration.

EtOH acts on diverse receptors within the brain with low affinity and specificity, affecting a variety of different functional pathways [1]. EtOH is distributed in the body similar to water, and its free diffusion through the blood-brain barrier explains its effect within 15–20 minutes of ingestion [1]. Low doses of EOH suppress brain activity, with reductions in glucose metabolism in the cerebellum and occipital cortex [2]. This effect may be mediated either by increased inhibition or reduced excitation. Although EtOH can suppress both NMDA and non-NMDA glutamatergic receptors, its main effect likely occurs by potentiation of inhibition of cerebral pathways [3]. EtOH is a GABAergic molecule, mainly targeting the GABA_A_ receptor–pentameric, ionotropic, ligand-gated chloride channels formed from different combinations of 19 possible subunits. In particular, α4β3δ and α6β3δ GABA receptors were showed to have a higher affinity for low doses of EtOH (i.e. few millimoles, as found in half a glass of wine), with important functional and topographical implications [4]. Indeed, the presence of β3-subunits are associated with higher sensitivity to EtOH, while the presence of δ-subunits, which in vivo are found almost only in combination with the α4 and α6 subunits, determines the extrasynaptic localization of these receptors [4,5,6,7]. Compared to other GABA receptors, those containing α6 and δ-subunits generate a tonic inhibition, instead of phasic inhibition [8]. Of note, α4β3δ and α6β3δ GABA receptors are preferentially expressed in the cerebellum, and to a lesser extent in the hippocampus, thalamus and frontoparietal cortex, and the α6-contaning GABA receptors are found almost only in the cerebellar granule cells [9]. Identification of the specific binding sites in these subunits may be relevant for designing drugs with selective EtOH effects and to explain variable responses to GABA receptor ligands related to specific polymorphisms in genes encoding for these subunits, as previously shown [10]. For example, the compound AA29504 has been reported to have specific affinity for the αβδ receptors [11]. On the other side, Purkinje cells are instead enriched with α1β2/3γ2 GABA receptors [12]. Interestingly, GABA_A_ receptors are the targets of other drugs, for example benzodiazepine (BZDs). Different from EtOH, these drugs bind specific BZD sites on GABA, particularly GABA_A_ receptors rich in γ subunits which are mostly synaptic [13,14]. GABA_A_ receptors are also the target of phenobarbital and primidone, however while EtOH and BDZ increase the frequency of receptor opening, phenobarbital acts by increasing duration of opening, with a possible different effect on downstream pathways [15]. In addition to acting as a GABA_A_ receptor agonist, EtOH increases GABA release in the granule cells of the cerebellum through inhibition of nitric oxide synthase [16]. Other receptors modulated by EtOH are glycine and adenosine where EtOH potentiates their inhibitory effect, as well as serotoninergic receptors and dopaminergic pathways [1].

PET studies have shown that patients with essential tremor (ET) treated with EtOH experience a reduction in cerebral blood flow in the cerebellum, and an increase in blood flow in the inferior olivary nucleus (ION) [17]. This observation suggests a possible mechanism by which EtOH might reduce cerebellar-driven tremor, by suppressing cerebellar cortex hyperactivation (which has an inhibitory effect on the deep cerebellar nuclei). EtOH increases inhibitory output from the cerebellum to the ION (and thus increased blood flow in this area); consequently, ION stimulation is reduced and tremor is suppressed [17]. Consistent with this idea, EtOH has also been reported to be a decoupling agent, able to inactivate gap junctions that are normally found in synaptic clefts and are particularly represented in the ION [18]. EtOH also exerts an interesting effect on the striatum, where it potentiates GABAergic activity in the dorsomedial striatum which controls goal-directed actions (“associative” striatum), and inhibits GABAergic activity in the dorsolateral striatum (“sensory-motor” striatum), which controls unconscious, automatic actions [19].

The effect of EtOH analogues on alcohol-responsive movement disorders has also been studied. Gamma-hyroxybutyric acid (GHB) is a derivative of GABA with similar effects to EtOH. GHB is found as an endogenous molecule within the brain, although at very low concentrations. Sodium oxybate (Xyrem), the sodium salt of GHB, has been studied as a potential treatment for refractory alcohol-responsive movement disorders. Like EtOH, GHB reaches a peak dose within 35 minutes of administration, and plasma levels show a direct, non-linear dose response. At higher doses the sedative effect peaks later (40 vs 60 minutes at a dose of 25 vs 35 mg/kg, respectively) and decays slower, reaching baseline in no more than 3 hours [20]. GHB binds with low affinity to the metabotropic GABA_B_ receptor, the target of the drug baclofen, as well as distinct high-affinity binding sites [21]. GHB’s interaction with GABA_B_ receptors likely occurs only with administration of exogenous GHB [22] given this low affinity. GABA_B_ receptors are expressed in the cortex, hippocampus, thalamus (especially in the ventroposterior lateral and medial thalamus responsible for the generation of oscillatory activities to the cortex), and cerebellum, particularly in the Purkinje cells [23,24]. Different studies report an increased expression of the high-affinity binding sites for GHB in the frontal cortex and hippocampus, and a lower expression in the cerebellum [25]. However, specific GHB receptors with low affinity have been identified in the cerebellum, especially in the Purkinje cells [26] (possibly missed by autoradiographic studies assessing the distribution of the high affinity binding sites). Different doses of GHB can target distinct receptors and pathways [27]. Interestingly, low doses of GHB (1 μM) are active on the α4β3δ GABA_A_ receptors, that, as reported above, are also the targets of low dose EtOH [28].

The complexity of the pharmacology of EtOH and GHB poses a significant obstacle to understanding the effectiveness of these agents in patients with alcohol-responsive hyperkinetic disorders. The pharmacokinetics and pharmacodynamics of EtOH are poorly explained by the binding characteristics of a single drug to one neurotransmitter receptor. A plausible model must explain the response of etiologically diverse disorders (posthypoxic myoclonus (PHM), ET and myoclonus-dystonia (MD)) to EtOH or GHB, and also explain the unique pharmacokinetics of this response: its rapid onset of action; the duration of benefit; its dose-dependence; and the lack of tachyphylaxis. Finally, a working hypothesis should be testable in animal models of these disorders, and in affected patients using functional imaging and neurophysiologic investigations.

## Literature Review: Alcohol-responsive Hyperkinetic Movement Disorders

We performed a Pubmed search using the following key words: alcohol, sodium oxybate, GHB, dystonia, tremor, chorea, tics, and myoclonus. All papers with reported response to EtOH or GHB (or its sodium salt, sodium oxybate, commercially available in the United States as the FDA-approved drug Xyrem) were reviewed. We refer to GHB and Xyrem interchangeably in the remainder of this paper, recognizing that patients treated in the United States received the drug Xyrem. We included all reported prospective studies or clinical observations, and also included historical descriptions of a response to EtOH when prospective observations were unavailable. The literature search identified the following hits: 468 papers for the terms “dystonia and alcohol” (5 were included in the manuscript); 309 papers for the terms “myoclonus and alcohol” (11 were included in the manuscript); 94 papers for the terms “tic and alcohol” (0 were included in the manuscript); 403 papers for the terms “chorea and alcohol” (0 were included in the manuscript); 2076 papers for the terms “tremor and alcohol” (11 were included in the manuscript); 11 papers for the terms “dystonia and GHB” or “dystonia and sodium oxybate” (1 was included in the manuscript); 12 papers for the terms “myoclonus and GHB” or “myoclonus and sodium oxybate” (9 were included in the manuscript); 9 papers for the terms “tremor and GHB” or “tremor and sodium oxybate” (1 was included in the manuscript); 0 papers for the terms “chorea and GHB” or “chorea and sodium oxybate” or “tics and GHB” or “tics and sodium oxybate”.

Table 1 lists fifteen alcohol-responsive hyperkinetic disorders, in which at least one patient in the literature responded to treatment with EtOH or GHB. Five tremor disorders responded to EtOH: essential tremor (ET), isolated vocal tremor (VT), primary writing tremor (PWT), orthostatic tremor (OT), and tremor in Kennedy’s disease [29,30,31,32,33,34,35,36,37,38,39,40]. Six alcohol-responsive dystonic disorders were reported: torticollis, abductor spasmodic dysphonia (ABSD), adductor spasmodic dysphonia (ADSD), ADSD in DYT-4 dystonia, dopa-responsive dystonia (DYT-5, DRD), and generalized dystonia [41,42,43,44,45,46]. Four alcohol-responsive myoclonic disorders have been reported: myoclonus-dystonia linked to epsilon sarcoglycan mutations (SCGE-MD), posthypoxic myoclonus (PHM), Unverricht-Lundborg disease (EPM-1), and sialidosis-type-1 [47,48,49,50,51,52,53,54,55,56,57]. Details of the published studies are summarized in Table 2.

**Table 1:** **Hyperkinetic movement disorders with reported response to EtOH or GHB**. Hyperkinetic movement disorders responsive to EtOH or GHB are listed in Table 1. Tremor disorders appear in green, myoclonic disorders in blue, and dystonic disorders in red.


Essential tremor (ET)
Isolated vocal tremor (VT)
Primary writing tremor (PWT)
Orthostatic tremor (OT)
Tremor in Kennedy’s disease (X-linked Spinal Bulbar Muscular Atrophy)
Myoclonus-dystonia linked to epsilon sarcoglycan mutation (SCGE-MD)
Posthypoxic myoclonus (Lance Adams syndrome, PHM)
Progressive Myoclonic Epilepsy type 1, (EPM1)
Adult sialidosis type I
Torticollis
Abductor spasmodic dysphonia (ABSD)
Adductor spasmodic dysphonia (ADSD)
Adductor spasmodic dysphonia in DYT-4
Dopa-responsive dystonia (DYT-5, DRD)
Generalized dystonia


**Table 2:** **Summary table of published studies of hyperkinetic movement disorders treated with EtOH or Xyrem**. Thirty-one published studies of hyperkinetic movement disorders treated with EtOH or GHB are presented in Table 2. Papers are grouped by diagnosis, with tremor disorders in green, myoclonic disorders in blue, and dystonic disorders in red. Columns, starting from the left, include: **Reference (reference #3)**; **Underlying diagnosis** (ET—essential tremor, PWT—primary writing tremor, VT—vocal tremor, OT-orthostatic tremor, X-SMA—X-linked spinal muscular atrophy, PHM—posthypoxic myoclonus, PME—progressive myoclonic epilepsy; MD—myoclonus dystonia, MG—Meige, SD—spasmodic dysphonia, WC—writer’s cramp, GD—generalized dystonia, RAS—Rasmussen’s, DRD—dopa-responsive dystonia); **n**—number of reported patients; **Selection of patients by response?**—whether or not patients were chosen based on their response to EtOH (selected) or not (unselected); **EtOH**—patients who received EtOH and those with concentration of EtOH measured (Yes) or not (No); **How achieved?**—Mechanism of administration of EtOH; **GHB**—those patients who received GHB, and dosing; **Placebo**—whether or not a placebo administration was employed (Yes) or not (No); **Measure/rating**—mechanism of rating of the response, including rating scale, accelerometer, WHIGET (Washington Heights Inwood Generalized Essential Tremor rating scale), observation, UMRS (Unified Myoclonus Rating Scale); **Effect size**—estimation of % of benefit, or verbal description; **t ratings**—time points at which improvement was measured; **t onset**—time at which first benefit was measured; **t max**—time at which maximum benefit was observed; **Dose response**—whether a dose response was observed (Yes), not observed (No), or not known (N/A); **Blinded rating**—whether ratings were blinded (Yes) or not (No); **Rebound**—whether rebound worsening was observed (Yes) or not (No) or unknown (N/A); and, **Tachyphylaxis**—whether this was observed (Yes), not observed (No) or unknown (N/A).

Reference [#]	Diagnosis	n	Selection of patients by response?	EtOH [ ]	How achieved?	GHB	Placebo	Measure/Rating	Effect size	t ratings	t onset	t max	Dose response?	Blinded rating?	Rebound?	Tachyphylaxis?

Hopfner [21]	ET	71	Unselected	Yes	Oral:Widmark		Yes	Blinded Archimedes rating	Arch spiral: 50%	0, 20’, 40’, 60’, next AM	20’	60’	N/A	Yes	Yes	N/A
Knudsen [22]	ET	25	Unselected	Yes	Oral:Widmark		No	Blinded spirals; Tremor scale	>50%	0, 10’20’, 30’, 40’, 50’, 60’, 90’, next AM	10’	40’	N/A	Yes	Yes	N/A
Voller 2014 [25]	ET	15	Unselected	Yes	Oral breathalyzer		No	Acceleromter/TETRAS	40%	0, 20’, 40’, 60’, 80’, 100’, 120’	20’	60’	N/A	No	N/A	N/A
Zeuner 2003 [26]	ET	10	Selected	Tes	Oral bolus		No	Accelerometer/performance	>50%	0, 30’, 60’, 90’, 120’	30’	60’	N/A	No	N/A	N/A
de Haas 2012 [27]	ET	9	Selected	Yes	IV infusion		Yes	Accelerometer/performance	>50%	0, 60’, 150’, 240’, 330’, 420’	60’	60’	N/A	No	N/A	N/A
Frucht 2005 [28]	ET	9	Selected			1–3 gm	No	WHIGET	50%	60’	45’	60’	Yes	Yes	N/A	No
Growdon 1975 [29]	ET	5	Unselected	Yes	Oral bolus		No	Accelerometer	>50%	q 10’	15’	15’	N/A	Yes	Yes	N/A
Bain 1995 [30]	PWT	7	Unselected	No	History		No	Observation	greatly improved or abolished	N/A	N/A	N/A	N/A	No	N/A	N/A
Koller [31]	PWT	1	Unselected	No	History		No	Observation	“improved”	N/A	N/A	N/A	N/A	No	Yes	N/A
Sulica [32]	VT	9	Unselected	No	History		No	Observation	“improved”	N/A	N/A	N/A	N/A	No	N/A	N/A
Massey [23]	VT	4	Unselected	No	History		No	Observation	“improved”	N/A	N/A	N/A	N/A	No	N/A	N/A
Dias 2011 [24]	X-SMA	7	Unselected	N/A	N/A		No	Observation	significant resposne	N/A	N/A	N/A	N/A	No	N/A	N/A
Frucht 2005 [28]	PHM	4	Selected			1–3 gm	No	UMRS	action myoclonus 45 →26	N/A	30’	60’	Yes	Yes	No	No
Frucht 2005 [39]	PHM	1	Selected	No	Oral	1–4 gm	No	UMRS	action myoclonus 108 →53	N/A	30’	60’	Yes	Yes	No	No
Frucht 2005 [40]	PHM	1	Selected			1–4 gm	No	UMRS	action myoclonus 108 →54	N/A	30’	60’	Yes	Yes	No	No
Arpsella [41]	PHM	1	Selected	Yes	IV infusion	2 gm	No	UMRS	action myoclonus 112 →44	N/A	N/A	60’	Yes	No	N/A	N/A
Riboldi 2019 [42]	PHM	1	Selected	No	Oral	1–2 gm	No	Video rating	marked improvement	60’	30’	60’	Yes	No	No	No
Jain 1991 [43]	PHM	1	Selected	No	Oral		No	Observation	dramatic improvement	30’; 60’; 240’	30’	30’	Yes	No	No	No
Lu 1991 [44]	PME	3	Selected	Yes	Oral		No	Observation	mild to dramatic improvement	20’	20’	20’	No	No	N/A	No
Frucht 2005 [28]	PME	2	Selected	No	Oral	1–3 gm	No	UMRS	moderate improvement	60’	30’	60’	Yes	Yes	No	No
Jain 1996 [45]	PME	2	Selected	Yes	Oral		No	Observation	disappearance of myoclonus	300–360’	20’	N/A	No	No	No	N/A
Genton 1992 [46]	PME	1	Selected	Yes	Oral		No	Observation	dramatic	within a few minutes	15’	N/A	No	No	N/A	Yes
Genton 1990 [47]	PME	4	Unselected	No	Oral		No	Observation	mild to dramatic improvement	N/A	10’	N/A	N/A	No	No	N/A
Weissbach 2017 [48]	MD	17	Selected	Yes	Oral breathalyzer		No	UMRS	Mean score: 37 →20	300–360’	20’	N/A	No	No	N/A	N/A
Frucht 2005 [40]	MD	2	Selected			1–4 gm	No	UMRS	action myoclonus 25 → 3; 35 → 15	60’	30’	60’	Yes	Yes	No	No
Frucht 2005 [28]	MD	1	Selected			1–4 gm	No	UMRS	moderate improvement	60’	30’	60’	Yes	Yes	No	No
Priori 2000 [49]	MD	1	Selected	No	N/A	1–5 gm	No	Observation	80% improvement	N/A	N/.A	60’	N/A	No	No	No
Rumbach 2017 [33]	SD, SD/VT	45	Selected		Oral	1–1.5 gm	No	Speech rating of recordings	33% improvement	45’	30’	45’	No	Yes	N/A	N/A
Wilcox 2011 [34]	SD	7	Unselected	No	Oral		No	Observation	mild to sigificant	N/A	N/A	N/A	N/A	No	N/A	N/A
Biary 1985 [35]	CD	7	Unselected	Yes	IV infusion		Yes	Rating Scale	Dystonia score: 63 → 36	N/A	15’	15’	N/A	Yes	N/A	N/A
Lim 2012 [36]	WC	1	Selected	Yes	Oral		No	Observation/physiology	near complete resolution	N/A	10’	10’	N/A	No	No	No
Micheli 2017 [37]	GD	1	Selected	Yes	Oral		No	Observation	dramatic improvement	N/A	rapid	N/A	N/A	No	No	No
Grantham 2014 [38]	DRD	1	Selected	Yes	Oral		No	Observation	complete resolution	N/A	rapid	N/A	N/A	No	No	No
**Totals**		***276***									***15–45’***	***≤60’***				

**Figure 1:**
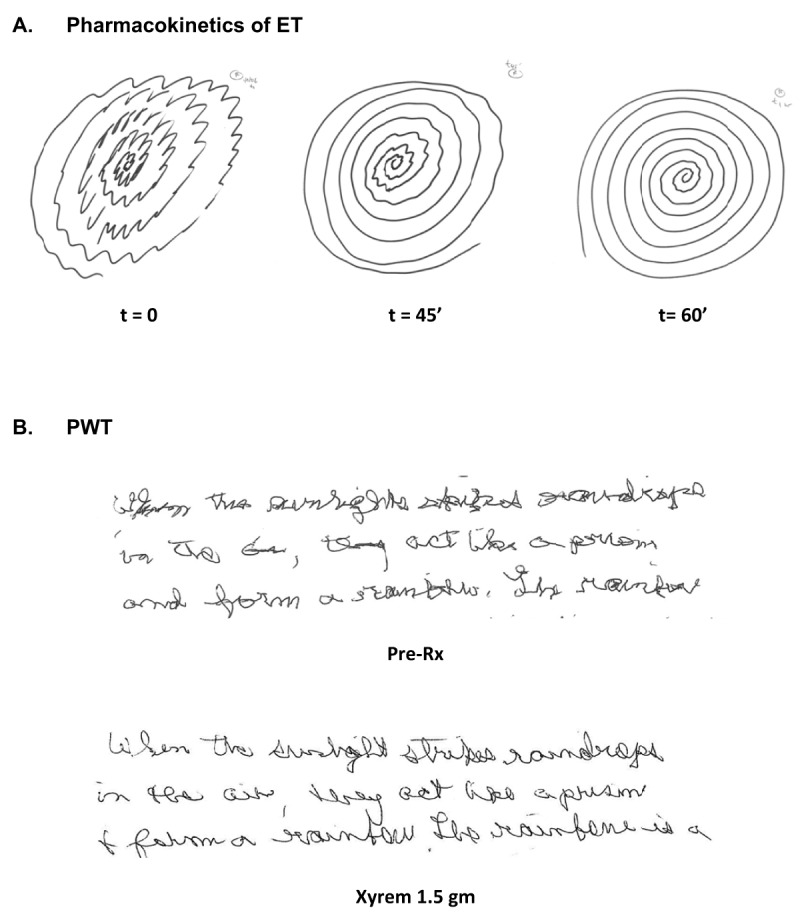
**Written examples of the effect of Xyrem on ET and PWT**. Written examples of the effect of Xyrem on ET and PWT appear in Figure 1. In Figure 1A, Archimedes spiral samples correspond to the video segment of patient #8 while she was filmed at fifteen minute intervals (t = 0, 15 min, 30 min, 45 min, 60 min) after receiving 1.5 gm of Xyrem. A classic ET spiral is seen at t = 0, with a characterstic axis of maximum amplitude of tremor of approximately 60 degrees. Forty five minutes later, the amplitude of the tremor is reduced, and tremor is nearly absent at sixty minutes. The frequency of the tremor is unchanged by treatment. Benefits in Archimedes spiral correlate with clinical benefits in pouring water demonstrated in patient #8’s video segment. Handwriting samlpes of a portion of the “rainbow passage” in a patient with PWT are diplayed before and one hour after treatment with 1.5 gm of Xyrem (Figure 1B). The improvement of writing tremor is only modest, but legibility is improved.

Several common themes emerge from review of these case series. Observed improvements with modest doses of EtOH or Xyrem are rapid, sometimes visible 15 minutes after the drug is administered, and always evident by 45–60 minutes. Response to treatment is typically dose-dependent, lasts three to four hours, and worsens the next morning with rebound in the case of EtOH. Tachyphylaxis to treatment with Xyrem was not seen, and evidence for the phenomena with EtOH is unavailable. Over the last fifteen years, we have conducted five IRB-approved clinical trials of Xyrem in patients with alcohol-responsive movement disorders, including patients with PHM, ET, VT, SCGE-MD, ADSD and ABSB. We have administered the drug to more than one hundred patients in clinical trials or as part of clinical care where other therapeutic options have failed. The response to EtOH appears to predict response to Xyrem, and the pharmacokinetics and tolerability of the two agents appear to be very similar. In the accompanying video segment and video legend, we present select patient responses to EtOH or sodium oxybate; these video segments illustrate robust improvements, understanding that mild or moderate improvements are more typical. Improvements in Archimedes spirals (ET) and handwriting (PWT) with administration of Xyrem appear in Figure 1.

## A new model

**Figure 2:**
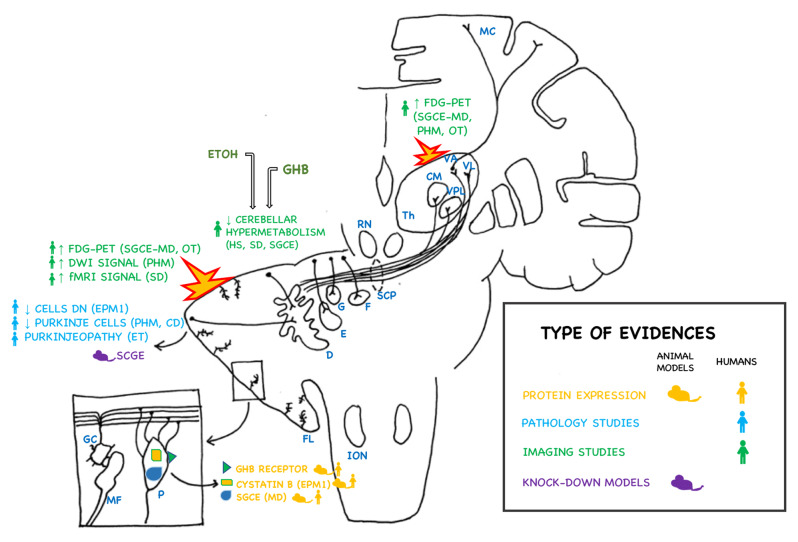
**Evidence supporting the hypothesis of the cerebellum and dentate nucleus in the pathogenesis of alcohol-responsive movement disorders**. Evidence from imaging studies, neuropathology, animal models, and molecular evidences (such as protein expression) are captioned in the figure. The topographic distribution of involved brain regions and structures are shown in the scehematic representation of the cerebral and cerebellar hemispheres. The source of the evidence in human subjects (human silhouette) or in animal models (mouse cartoon) is also depicted. Different types of evidences are color coded (yellow: molecular studies – protein expression; light blue: pathology studies; green: imaging studies; purple: animal models). Activated areas (cerebellar cortex and thalamus) in these disorders are highlighted in the figure. ION: inferior olivary nucleus; FL: flocculonodular lobe; D: dentate nucleus; G: globose nucleus; E: emboliform nucleus; F: fastigial nucleus; SCP: superior cerebellar peduncle; RN: red nucleus; Th: thalamus; CM: centromedian nucleus; VPL: ventral posterolateral nucleus; VL: ventral lateral nucleus; MC: motor cortex; MD: myoclonus-dystonia; EPM1: Progressive myoclonic epilepsy type 1; ET: essential tremor; PHM: post-hypoxic myoclonus; CD: celiac disease; SD: spasmodic dysphonia; OT: orthostatic tremor; HS: healthy subjects; FDG-PET: fluoro-deoxy-glucose positron emission tomography. In the box in the left side corner: a schematic of magnification of the cellular structure of cerebellar cortex (P: Purkinje cell; MF: mossy fiber; GC: granular cells).

We propose a new model to explain the phenomenon of alcohol-response in select hyperkinetic movement disorders. A diagram illustrating this model and its supporting evidence appears in Figure 2.

*We propose that the improvement of varied hyperkinetic movement disorders with modest doses of EtOH or GHB does* ***not*** *derive from a simple pharmacologic effect on the GABA-A, GABA-B or GHB receptors. Instead, we propose that* ***modest doses of GHB or EtOH possess a specific and novel ability to normalize pathologic hypermetabolism of the cerebellar Purkinje cells and deep cerebellar nuclei*.** *We further propose that* ***Purkinje cell dysfunction*** *(either aberrant activation or abnormal synchronous firing)* ***is the unifying feature linking these varied hyperkinetic disorders***.

## Evidence

We present two lines of evidence to support this hypothesis:

### A: Evidence of the effect of modest doses of EtOH or GHB on cerebellar metabolism

In a series of three papers, Volkow and colleagues investigated the effect of a modest dose of EtOH in normal individuals, employing doses that were not intoxicating or sedating. In both women and men, a single low dose of EtOH produced the greatest metabolic reduction in the cerebellum, with no change in thalamic metabolism and a mild increased metabolism in striatum [58]. A second study using increasing modest doses of alcohol [59] again showed the largest metabolic reductions in the cerebellum as well as thalamus and mesencephalon. The final study with co-registered MRI localization confirmed this effect on the cerebellum and occipital cortex [60]. These three papers illustrate that modest doses of EtOH selectively and preferentially reduce cerebellar metabolism. To our knowledge, the effect of a modest dose of GHB on cerebral metabolism in man or in animals has not been studied. Interestingly, in rat brain the GHB receptor is heavily expressed in the cerebellum but not in striatum or thalamus, and within the cerebellum GHB-receptor expression is highest within Purkinje cells [61]. Taken together, these three pivotal studies support the idea that administration of EtOH at doses that do not produce intoxication or sedation selectively reduces cerebellar metabolism.

### B: Evidence of the role of the cerebellum in alcohol-responsive movement disorders and its modulation by EtOH and GHB

Two post-mortem studies of coeliac disease patients with cortical myoclonus have demonstrated selective loss of Purkinje cells, illustrating that isolated cerebellar pathology can generate cortical myoclonus [62,63]. In EPM-1 (Unverricht-Lundborg disease), another disorder with prominent cortical myoclonus and EtOH-response, a post-mortem study showed a similar loss of Purkinje cells with involvement of the dentate nucleus [64,65]. Many patients with EPM1 do not appear to have cerebellar atrophy on routine MRI imaging, but an MRI/MRS study of a cohort of patients demonstrated mild atrophy of cerebellar hemispheres, medulla and basis pontis [66]. Cystatin B, the protein affected by EPM1, is selectively expressed in Purkinje cells and some molecular layer neurons in the developing and adult rat [67]. In man cystatin B expression is limited to Purkinje cells and Bergmann fibers [67]. The cerebellum has also been demonstrated to be critically important in PHM. In the best available animal model of PHM, Walsh demonstrated that circulatory arrest for eight minutes selectively injures bands of Purkinje cells, and is likely the signature lesion of PHM [68]. PET study of patients with PHM demonstrated a metabolic topographic pattern of activation of the VL thalamus and pontine tegmentum [69]. Re-analysis of this data and comparison to PET studies in SCGE-MD patients revealed hypermetabolism of the cerebellar cortex and dentate [70]. Finally, a single patient with PHM demonstrated transient increased DWI signal in the cerebellum and thalami, and these signal abnormalities remitted as the patient’s myoclonus subsided [71]. Taken together, these studies in animal and man of coeliac, EPM1 and PHM demonstrate a central role of the cerebellum and Purkinje cells in the generation of myoclonus.

Investigations in patients with SD and SCGE-MD support a pivotal role of the cerebellum and Purkinje cells in these disorders. In an fMRI study of SD patients treated with a single dose of Xyrem, clinical improvement in dysphonia and reduction in vocal breaks correlated with normalization of cerebellar activation [72]. In a PET study, symptomatic SCGE-MD patients demonstrated activation of cerebellar cortex and dentate compared to non-manifesting SCGE carriers and healthy controls [70]. In a post-mortem study of SCGE-MD patients, the brain-specific isoform of SCGE (exon 11b) was found to be highly expressed in Purkinje cells and dentate nucleus [73]. Finally, an elegant selective knockdown model of SCGE in the cerebellum of adult mice produced a robust MD phenotype [74]. SCGE was expressed in Purkinje cells and deep cerebellar nuclei, and administration of EtOH normalized output from these structures in SCGE knockdown mice but not in DYT-1 knockdown mice, illustrating their specific role in MD [74]. Taken together, these studies support the critical role of the cerebellum, Purkinje cells and dentate in MD, and the likely mechanism of action of EtOH to normalize Purkinje cell and cerebellar output in this disorder.

Many studies in the last fifteen years have demonstrated the important role of the cerebellum in ET, and it is beyond the scope of this paper to review this evidence in detail. Briefly, pathologic changes in post-mortem tissue support Louis’ designation of ET as a “Purkinjeopathy”, with a loss of Purkinje cells up to about 30% [75,76]. While no animal model fully replicates all of the clinical features of ET, Broersma demonstrated that ET tremor is correlated with bilateral cerebellar activation in lobules V, VI and VIII [77]. Pedrosa demonstrated that the effect of EtOH on ET tremor is due to normalization of cerebellar activation [78].

## Conclusion and unanswered questions

We have proposed a model to explain a robust and reproducible improvement with modest doses of EtOH or GHB in certain hyperkinetic movement disorders. We posit that abnormal activation of the Purkinje cells and dentate nucleus, the major outflow of the cerebellum, are critical to this response. Purkinje cells are involved pathologically in coeliac disease, ET, and PHM, and expression of cystatin B, SCGE 11b, and the GHB receptor are selectively increased in Purkinje cells. Network studies in ET, PHM, MD and SD support the role of abnormal cerebellar activation in these conditions. Granule cells in the cerebellum can be implicated as well. Indeed, GABA receptors sensitive to low doses of EtOH and GHB are specifically found on these cells. A primary disfunction in the Purkinje cells due to cell loss or protein abnormalities, such as cystatin B in EPM1, could cause abnormal firing of these cells. Modulation of the inputs from the Granule cells could possibly restore the cerebellar hyperactivity. Normalization of this network by EtOH (ET, MD), and Xyrem (SD) has been shown to be associated with clinical improvement. We demonstrate by **video** our experience over the last fifteen years treating patients with these conditions, and illustrate the rapid and significant effects of EtOH and Xyrem at modest doses.

**Video:** **Alcohol-responsive movement disorders—a unifying hypothesis?** We present video examples of robust responses to EtOH or Xyrem in thirteen selected patients treated by the senior author in IRB-approved clinical trials or clinical practice over the last fifteen years. We specifically selected video segments that illustrated a robust response. **Patient #1**, a 37-year-old woman, underwent a routine gynecological surgery complicated by an unrecognized esophageal intubation leading to refractory severe PHM [47]. Despite treatment with clonazepam, valproic acid, phenobarbital, topiramate, zonisamide and levetiracetam, paroxysms of myoclonus affecting the trunk, head and limbs, are triggered by any attempt to move. Twenty minutes after ingesting two eight-ounce glasses of wine in the office, her myoclonus improved for the first time in three and a half years, enough for her to gesture fluidly (telling her husband to “shut up”). Her husband was deeply moved, stating that “the gesture has returned”. She was even able to walk with only mild support from her home aide while the EtOH effect lasted. She participated in a single patient, IRB-approved, add-on clinical trial of Xyrem, and brief clips of her attempts to pour water are shown before and one hour after ingesting 4 gm of Xyrem (although she tolerated this dose without sedation, in subsequent trials lower doses of Xyrem were employed), After the trial concluded, she was treated with Xyrem in open label fashion for a decade at doses of 1.5 gm every three hours, until her demise from medical illness. **Patient #2** sustained an asthmatic arrest leading to PHM fifteen years before this video was taken. Despite treatment with clonazepam and levetiracetam, action and intention myoclonus and negative myoclonus on standing were significant. The video segment illustrates myoclonus before and one hour after ingestion of 2.5 gm of Xyrem [48]. **Patient #3** developed severe PHM after a spontaneous bilateral pneumothorax leading to cardiopulmonary arrest. Despite treatment with valproic acid, levetiracetam and zolpidem, severe action and intention myoclonus were disabling. He was admitted to hospital in order to titrate increasing doses of Xyrem in an observed setting (he did not receive an EtOh challenge as he was only 19 years old). One hour after administration of 1.5 gm of Xyrem, action and intention myoclonus were reduced, allowing him to perform tasks such as brushing his hair for the first time. He has remained on Xyrem for the last three years with clear awareness of kinetics of the drug, and no evidence of tachyphylaxis [51]; bilateral DBS of the GPi was performed two years after this video was taken, with additional functional benefit. **Patient #4** developed severe PHM after a cardiac arrest triggered by a pulmonary embolus. Despite treatment with clonazepam, valproic acid, zonisamide and levetiracetam, severe myoclonic jerks of his arms and torso left him completely functionally dependent. In this home video before and one hour after ingestion of six ounces of 80 proof vodka, significant improvement in myoclonus at rest and with action is evident. He did not tolerate Xyrem due to worsening depression, and he subsequently underwent bilateral DBS of the GPI, with surgical results pending at the time of this writing. **Patients #5–8** demonstrate the response of VT and ET to treatment with Xyrem in IRB-approved clinical trials [18,43]. **Patient #5**, a 61-year-old woman with VT, is shown speaking and phonating before and one hour after ingesting one gram of Xyrem. A moderate-amplitude vocal tremor is evident before treatment, with modest reduction in the amplitude of tremor (without change in frequency). **Patients #6–8**, all with ET, are shown in brief video clips before and after treatment with Xyrem [36]. **Patient #6** attempts to draw an Archimedes spiral with disastrous results; one hour after ingesting two grams of Xyrem he is able to perform the task. **Patient #7** is shown before and one hour after administration of 1.5 gm of Xyrem. Interestingly, the video shows that after treatment she was aware that she could pour water with her left hand before she attempts to perform the task. **Patient #8** was videotaped in fifteen-minute intervals after ingesting 1.5 gm of Xyrem to assess the pharmacokinetics of the improvement. Before treatment, action tremor of the right hand interferes with her attempt to pour water. Forty-five minutes after ingesting 1.5 gm of Xyrem, a significant reduction of tremor is seen, and tremor disappears at sixty minutes, surprising the patient and her husband. Despite this robust response, she did not continue treatment due to the sedative side effects of the drug. The following three patients with SCGE-MD **(#s 9, 10 and 11)** are shown in brief clips taken during their participation in a clinical trial [36]. **Patient #9** is shown pouring water before and one hour after administration of 2.5 gm of Xyrem. **Patient #10** is more severely affected, with myoclonus affecting walking and pouring. Myoclonus was moderately improved at relatively high doses of Xyrem (video shown one hour after administration of four grams). The final patient was afflicted with predominant axial jerks triggered by actions such as pouring. One hour after administration of two gm of Xyrem, myoclonus was improved. The final two **patients, #s 12** and **13**, participated in a study of the effects of Xyrem on SD with functional MRI [72]. **Patient #12** is afflicted with ADSD and is usually treated successfully with botulinum toxin injections bilaterally to the thyroarytenoid muscles. Her ADSD was exquisitely responsive to EtOH, and she is shown before and one hour after administration of 1.5 gm of Xyrem with near resolution of vocal breaks. The final patient, **patient #13**, is afflicted with ABSD, and is shown before and one hour after administration of one gram of Xyrem, with resolution of his abductor breaks.

Perhaps the biggest obstacle to our model is the question of how modest doses of EtOH or Xyrem exert their selective effect on the cerebellum. Selective knock-down and optogenetic studies might allow investigation of this question, and high-resolution MRI and co-registered PET studies in patients and animal models would also be useful. It is also possible that the nature of Purkinje cell dysfunction differs in the various disorders. Coeliac disease and anoxia selectively injure a subset of Purkinje cells, perhaps resulting in hyperexcitability in the remaining cells. In contrast, Purkinje cell dysfunction without overt cell loss may underlie the genesis of ET and MD. Given the robust nature of the EtOH and GHB response in this group of patients, further work to understand these phenomena and to design better therapeutic options is warranted.
